# Effect of the Mass Conversion Degree of an Oxygen Carrier on Char Conversion and Its Implication for Chemical Looping Gasification

**DOI:** 10.1021/acs.energyfuels.2c00944

**Published:** 2022-06-13

**Authors:** Victor Purnomo, Daofeng Mei, Amir H. Soleimanisalim, Tobias Mattisson, Henrik Leion

**Affiliations:** † Division of Energy and Materials, Department of Chemistry and Chemical Engineering, Chalmers University of Technology, Göteborg 412 58, Sweden; ‡ Division of Energy Technology, Department of Space, Earth, and Environment, Chalmers University of Technology, Göteborg 412 58, Sweden

## Abstract

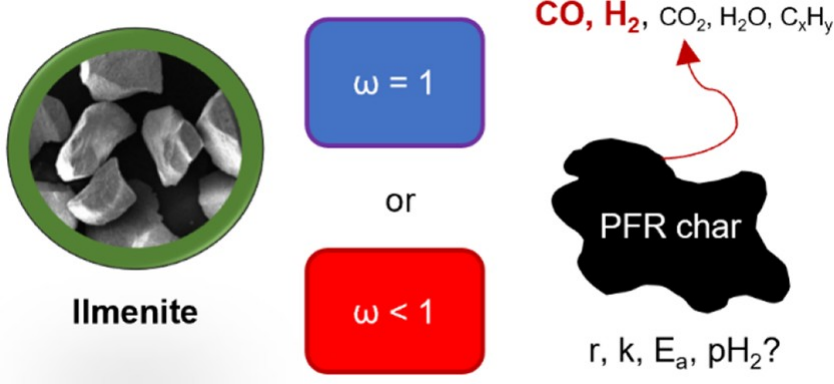

Chemical looping gasification (CLG) is an emerging process that aims to produce valuable chemical feedstocks. The key operational requirement of CLG is to limit the oxygen transfer from the air reactor (AR) to the fuel reactor (FR). This can be accomplished by partially oxidizing the oxygen carrier in the AR, which may lead to a higher reduction degree of the oxygen carrier under the fuel conversion. A highly reduced oxygen carrier may experience multiple issues, such as agglomeration and defluidization. Given such an interest, this study examined how the variation of the mass conversion degree of ilmenite may affect the conversion of pine forest residue char in a fluidized bed batch reactor. Ilmenite was pre-reduced using diluted CO and then underwent the char conversion at 850, 900, 950, and 975 °C. Our investigations showed that the activation energy of the char conversion was between 194 and 256 kJ/mol, depending upon the mass conversion degree of ilmenite. Furthermore, the hydrogen partial pressure in the particle bed increased as the oxygen carrier mass conversion degree decreased, which was accompanied by a lower reaction rate and a higher reduction potential. Such a hydrogen inhibition effect was confirmed in the experiments; therefore, the change in the mass conversion degree indirectly affected the char conversion. Langmuir–Hinshelwood mechanism models used to evaluate the char conversion were validated. On the basis of the physical observation and characterizations, the use of ilmenite in CLG with biomass char as fuel will likely not suffer from major agglomeration or fluidization issues.

## 1 Introduction

The greenhouse gases accumulating in the atmosphere are a relevant global issue. Emission-wise, conventional energy conversion processes, such as combustion, normally release a significant amount of greenhouse gases, e.g., carbon dioxide, to the atmosphere. The accumulation of greenhouse gases contributes to global warming and climate change, which are a growing concern to the environment and sustainability. Therefore, there is an urgency to establish a system that can convert fuel to other forms of energy without releasing the greenhouse gases to the atmosphere. One of the sound concepts to this need is the chemical looping process, which comprises chemical looping combustion (CLC) and chemical looping gasification (CLG), to name a few. Contrary to a conventional combustion or gasification setup, the system consists of two interconnected reactors, which make it possible to have nitrogen unmixed with the product gases in the outlet. In this way, the need for an expensive air separating unit after the fuel converter can be eliminated.

**Figure 1:**
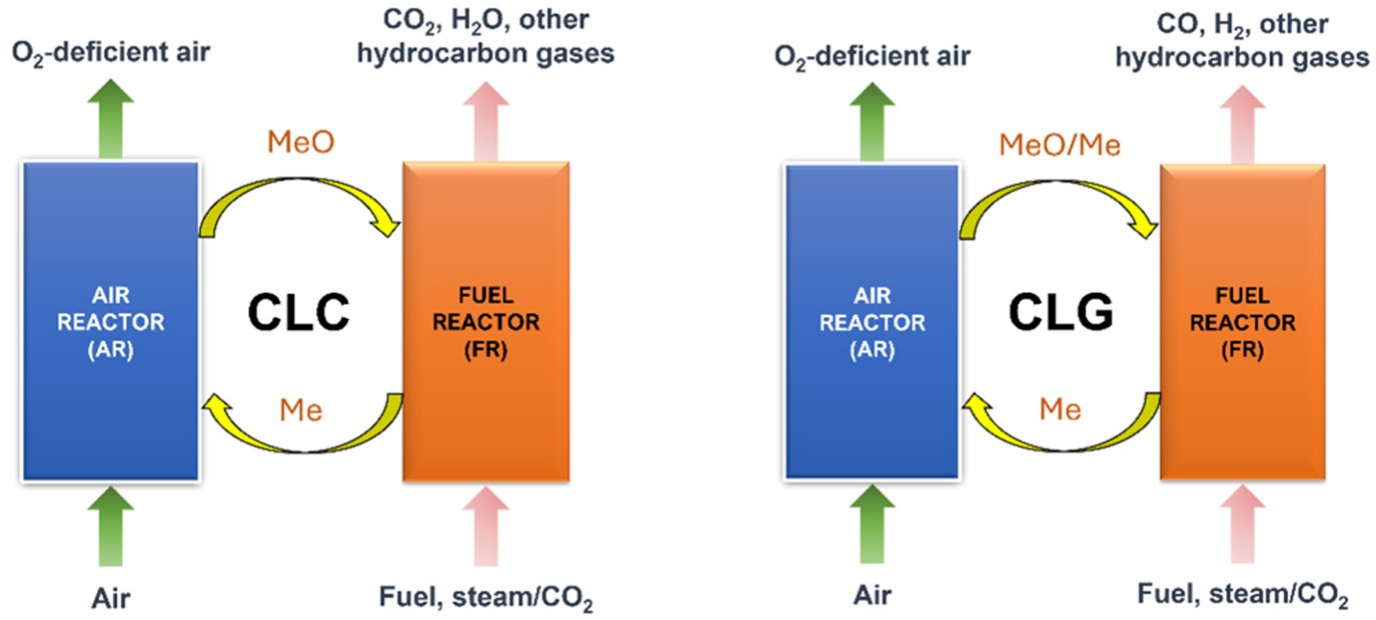
Schematic diagrams of CLC and CLG processes. MeO and Me represent fully oxidized and reduced oxygen carriers, respectively.

Figure 1 shows a general illustration of both CLC and CLG processes. In both setups, the fuel conversion taking place in the fuel reactor does not involve nitrogen. This is because oxygen in air is adsorbed by the oxygen carrier in the air reactor (AR), thus being separated from nitrogen and transported to the fuel reactor (FR) through circulation. Because the flue gas coming out from the fuel reactor only contains various hydrocarbons and water, the separation necessary for carbon capture can be performed through a simple condensation rather than in an expensive gas separation system.^1^ The difference between CLC and CLG lies mainly on the fuel conversion in the FR. CLC requires full oxidation of the fuel, thus producing mainly CO_2_ and H_2_O as the gaseous products. On the other hand, CLG maintains a partial oxidation of the fuel in the FR, thereby forming mainly CO and H_2_. While the main advantage of CLC is its reaction heat that can be used for heat-requiring processes and human activities, the focus on the CLG is the gaseous products themselves.

With CLG, it is possible to obtain a high-grade synthesis gas, which comprises carbon monoxide and hydrogen. These products can later be used to derive many useful liquid fuels through Fischer–Tropsch synthesis.^2^ The strong point of this type of gasification compared to a more conventional indirect gasification is that all carbon is released from the fuel reactor. CO_2_ will necessarily be produced in the process to fulfill heat balance, and because this is concentrated only to the FR, this opens up an efficient process for syngas production and, simultaneously, carbon dioxide capture. Given this emerging interest for an environmentally friendly fuel conversion, it is expected that the adaption of CLG is to convert biomass, an abundantly available carbon-neutral fuel, into valuable products. A review by Nguyen et al.^3^ stated that CLG has a clear advantage compared to the conventional gasification in terms of energy sourcing. While the conventional gasification requires a lot of energy, this issue is solved in CLG because the oxidation of the oxygen carrier taking place in the AR is exothermic. Not only is an oxygen carrier able to transport oxygen, but it can also transfer heat at the same time to the FR, facilitating an autothermic operation of the system. Lin et al.^4^ provided a comprehensive review of biomass chemical looping gasification (BCLG), suggested the use of certain types of biomass and oxygen carriers, and pointed out the potential to obtain syngas from CLG as a highly valuable chemical feedstock. Condori et al.^5^ studied BCLG using ilmenite in a 1.5 kW chemical looping unit with a notably successful control of oxygen fed to the AR. A simple process analysis predicted a high suitability of CLG for syngas production with a carbon capture process.^6^ Marx et al.^7^ pointed out that the cost-saving advantage of a dual-bed gasifier like CLG is the elimination of the air separation unit (ASU), which requires a lot of energy supply and high capital and operational costs.^8^

One of the key operation requirements in CLG is to maintain the partial oxidation of the fuel, where the oxygen transfer from the oxygen carrier to the fuel needs to be controlled.^9^ This can be done by maintaining a partial oxidation of the oxygen carrier in the AR by, for instance, lowering the oxygen partial pressure in the AR. Therefore, it is expected that the oxygen carrier will undergo a further reduction compared to that in CLC during the fuel conversion. This can significantly affect the performance of the oxygen carrier; e.g., the oxygen carrier may agglomerate and even defluidize under a highly reducing environment.^1^ An increase of iron content in a reduced state can enhance the attrition and degradation of the oxygen carrier particles, thereby shortening their lifetime.^5^ Cho et al.^10^ found that the further reduction of magnetite to wüstite can contribute to agglomeration and defluidization. In addition to these findings, we expected that a further reduction of the oxygen carrier might have affected the fuel conversion as well. Therefore, it is essential to study how a higher reduction degree may affect the CLG performance, particularly on the oxygen carrier properties and fuel reactivity.

The interest of study of kinetics of solid fuel conversion, including gasification, when the oxygen carrier is used as the fluidized bed has emerged. Haus et al.^11^ reported the kinetics of gasification of lignite char toward a copper-based oxygen carrier. Guo et al.^12^ studied the kinetics of gasification of three coal chars toward iron-based oxygen carriers at pressures between 0.1 and 1.2 MPa. Xu and Song^13^ used rice husk char and red mud oxygen carrier, a solid waste from an alumina-roasting process, in a CO_2_ gasification. Despite these, none of the previous studies has reported the kinetics of char conversion toward pre-reduced oxygen carriers, while this is highly applicable for CLG.

It has been known that, in any char conversion, the Arrhenius rate constants *k* and activation energy *E*_a_ are important parameters. In this case, the gas–solid reaction involving the oxygen carrier can be assumed to follow the first-order reaction.^14^ Furthermore, hydrogen partial pressure may inhibit the char conversion rate substantially. The hydrogen inhibition effect on the char conversion can be interpreted by the Langmuir–Hinshelwood equation, which considers adsorption–desorption mechanisms on the char surface that is relevant in a steam char gasification.^15^

In this study, ilmenite as an oxygen carrier was first pre-reduced to several mass conversion degrees prior to the conversion of pine forest residue char. The conversion rate and defluidization of the bed particle were monitored. The Arrhenius rate constant *k*, activation energy *E*_a_, and reduction potential are reported here. The char conversion kinetics and inhibition mechanisms were evaluated using Langmuir–Hinshelwood mechanism models. The physical and chemical properties of ilmenite, both before and after experiments at fully oxidized and reduced states, respectively, were characterized using X-ray diffraction (XRD) and scanning electron microscopy with energy-dispersive X-ray spectroscopy (SEM/EDX).

## 2 Experimental Section

### 2.1 Solid Fuel

Pine forest residue (PFR) as the biomass-based solid fuel was first degassed in an inert atmosphere (nitrogen) at 950 °C for 2 min in multiple batches of 5 g each. This was performed to remove the moisture and volatile contents, which comprise around 80 wt % of the char, that may affect the evaluation of char conversion in the batch reactor experiments. The obtained PFR char was subsequently crushed and sieved to the size range between 125 and 500 μm. The composition of the char is provided in Table 1. The moisture content observed in the char was likely due to the long-term storage of the char prior to the time when the analysis was carried out. The dry composition refers to the composition of the char that is dried once more after the storage.

**Table 1:** Composition of PFR Char

element	content (wt %)
total moisture	5.3
ash	14.1
chlorine (Cl)	0.01
sulfur (S)	0.05
carbon (C)	75
hydrogen (H)	1.1
nitrogen (N)	0.4

ash element	content (wt %, dry)
aluminum (Al)	0.45
silicon (Si)	1.90
iron (Fe)	3.90
titanium (Ti)	0.18
manganese (Mn)	0.22
magnesium (Mg)	0.31
calcium (Ca)	1.90
barium (Ba)	0.03
sodium (Na)	0.07
potassium (K)	0.63
phosphorus (P)	0.10

### 2.2 Oxygen Carrier

Ilmenite as the oxygen carrier used in this study was produced by Titania A/S in Norway. Ilmenite has been used in pilot-scale CLC reactors at different scales and even a commercial fluidized bed boiler with an oxygen carrier combustion process; thus, the material is deemed as the benchmark oxygen carrier.^16−20^ Vigoureux et al.^21^ presented the composition of ilmenite used in this study, which mainly comprises of 34.2% Fe and 27.9% Ti. Ilmenite was primarily calcined at 950 °C for 12 h in a high-temperature oven to obtain the oxygen carrier at a fully oxidized state and improve its reactivity to some extent.^22^ The oxygen carrier was then sieved to the size range between 125 and 180 μm.

### 2.3 Fluidized Bed Batch Reactor

**Figure 2:**
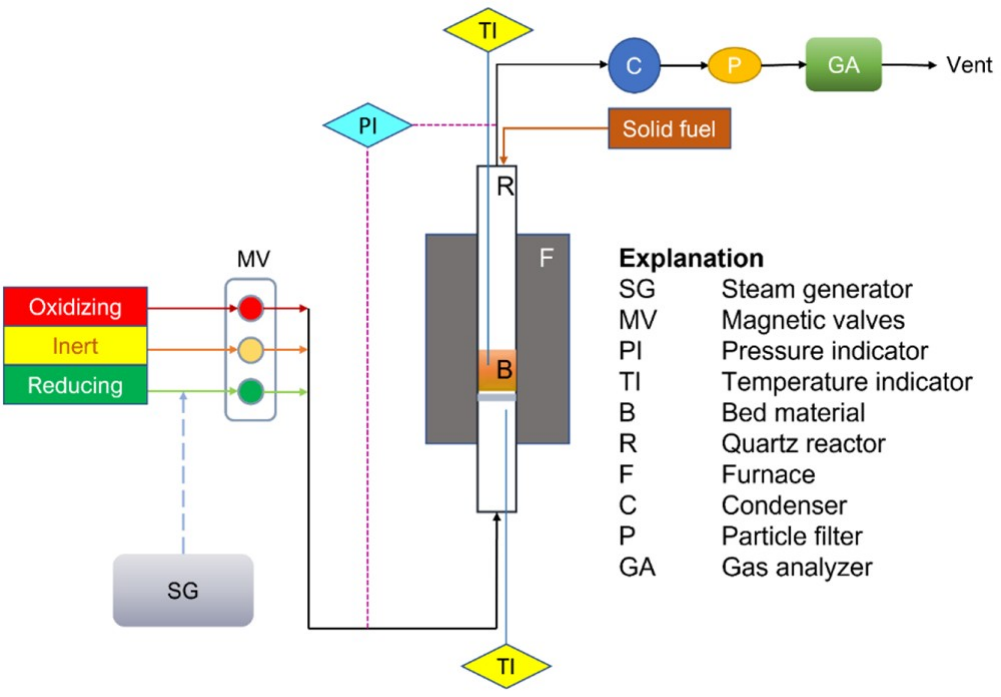
Schematic setup of the fluidized bed batch reactor system.

The experiments were conducted in a fluidized bed batch reactor that was heated in a high-temperature furnace. The schematic setup is illustrated in Figure 2.

The feeding of gases, either oxidizing, inert, or reducing, is regulated by the magnetic valves. The solid fuel is inserted from the top, while the gaseous fuel is injected from the bottom. The height and inner diameter of the straight quartz glass reactor were 820 and 22 mm, respectively. A porous quartz particle bed holder was placed 370 mm above the bottom edge of the reactor. The reactor was heated inside a furnace manufactured by ElectroHeat Sweden AB with gastight connections on its upper and lower parts. The top and bottom of the reactor were each wrapped with a heating tape to avoid outlet gas and steam condensation, respectively. A pair of type K thermocouples measured the real-time temperature inside the particle bed and below the bed holder; the temperature read by the former is used as the reference. A M&C ECP1000 cooler was installed downstream to remove water before entering the Rosemount NGA 2000 gas analyzer, in which gas flow volumetric rates and concentrations of CH_4_, CO, CO_2_, H_2_, and O_2_ were measured. The controlled evaporator mixer Bronkhorst-type W-202A-300-K was used to generate the steam. Pressure drops over the inlet and outlet of the reactor was regularly registered and used to monitor the fluidization state of the bed. To monitor the fluidization of the bed particle, a 20 Hz Honeywell pressure transducer was installed to measure the pressure difference between the inlet and outlet of the reactor. The frequency was deemed sufficient to judge the fluidization status of the bed particle.^1^ The setup was designed by Leion et al.^23^

### 2.4 Experimental Procedure

The conversion of PFR char was performed at four temperatures: 850, 900, 950, and 975 °C. The mass of the ilmenite particle bed was 20 g. Ilmenite was first activated at 850 °C for at least 3 cycles prior to the experiments to obtain a stable fuel conversion^24^ using diluted carbon monoxide (50% CO and 50% N_2_). Fully oxidized and partially reduced ilmenite were employed in the investigations. The mass conversion degrees of partially reduced ilmenite were set by exposing ilmenite to the same gaseous fuel, i.e., diluted carbon monoxide, for certain durations in the fluidized bed reactor prior to the char conversion (see Table 2). These durations translate to different mass conversion degrees, which will be provided later in the Results and Discussion. The pre-reduction of ilmenite was performed using diluted CO because it was easier to determine the mass conversion degree using gaseous fuels, instead of solid fuels, which have more complex composition. Steam and nitrogen were introduced into the reactor for 2 min before the char feeding. This was performed to make sure that the steam entered the reactor and came into contact with the ilmenite particle bed. PFR char was inserted to the reactor as a single batch of 0.1 g for every cycle, pushed by the continuous sweep gas nitrogen. The reaction was stopped when no more carbon conversion could be observed. Table 2 shows the complete procedure of a cycle in the study. All of the experiments were performed at atmospheric pressure, which was assumed as 1 atm. Ilmenite was exposed to 60 cycles in total. Dependent upon the temperature, the minimum fluidization velocity in the fluidized bed was between 0.66 and 0.71 cm/s, while the superficial fluidization velocity was between 10 and 11 cm/s.

**Table 2:** Complete Procedure in a Cycle

step	duration (s)	material and gas	amount
oxidation	until the oxygen carrier is fully oxidized	5% O_2_ in N_2_	600 mL/min
inert	180	pure N_2_	600 mL/min
diluted CO injection (pre-reduction)	0, 50, 100, 150, and 200	50% CO in N_2_	600 mL/min
inert	180	pure N_2_	600 mL/min
solid fuel conversion	until no more carbon conversion was observed	solid fuel: PFR char	0.1 g
		sweep gas: pure N_2_	300 mL/min
		fluidizing gas: 50% steam in N_2_	600 mL/min
inert	180	pure N_2_	600 mL/min

Apart from the procedure provided in Table 2, additional experiments were performed by (i) prolonging the pre-reduction durations of the ilmenite bed using diluted CO and (ii) injecting hydrogen with various concentrations to the sand bed. The motivation of this additional effort was to increase the hydrogen partial pressure in the particle bed because the experiments where only steam and nitrogen were used produced rather low concentrations of hydrogen. Because prolonging the pre-reduction duration using CO was not enough to increase the hydrogen partial pressure substantially, sand bed was latter used instead of ilmenite. The use of sand bed can avoid a further conversion of the additional injected hydrogen to steam, thus maintaining hydrogen partial pressure in the bed. These experiments are provided in Table 3.

**Table 3:** Additional Experimental Scheme at 900 °C

type of additional experiment	bed material	parameter (unit)	variable
longer pre-reduction with diluted CO	ilmenite	duration of diluted CO injection (s)	250, 300, 350, and 400
hydrogen injection	sand	hydrogen inlet concentration (%)	0, 5, 10, 15, and 20

### 2.5 Data Evaluation

The mass conversion degree of ilmenite with respect to carbon monoxide were evaluated on the basis of eq 1.
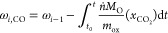
1Char conversion was calculated as the total detectable released carbon, which refers to the sum amount of all of the carbon-based compounds divided by the total emitted carbon detected under the whole period of the fuel conversion.^25^
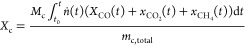
2The fuel conversion rate in a fluidized bed may fluctuate. On the basis of the previous study by Azimi et al.,^26^ the stable conversion rate was observed between the char conversion of 30 and 70%. The char conversion rate *r* can be expressed as a function of the fraction of char conversion at a specific time, *X*_c_, as seen in eq 3. With integration of both sides, the equation can be transformed to a linear correlation, which is shown in eq 4.^27^ In this study, the char conversion rate was obtained as the arithmetic mean of the gas–solid reaction rates over 3 repeated cycles.
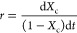
3
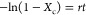
4The char conversion rate can be expressed as a function of the temperature, as shown in eq 5, which is based on the Arrhenius equation. The expression can be integrated to eq 6, which was plotted to find the kinetic rate constant, *k*, and activation energy, *E*_a_.
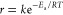
5
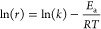
6

### 2.6 Char Reactivity Models

The experimental results were evaluated according to Langmuir–Hinshelwood models, which, as mentioned above, have previously been proven suitable to the steam gasification of char that involves a mixture of CO, CO_2_, H_2_, and H_2_O (steam).^15^ The aim of using the models is to examine the effect of hydrogen inhibition on the char conversion. Previous studies found that the hydrogen inhibition effect has been found to be more dominant than the negligible CO inhibition in a char steam gasification.^26,15^ Three model mechanisms have been used by Azimi et al.,^26^ which are oxygen exchange (OE), associative hydrogen adsorption (AHA), and dissociative oxygen adsorption (DHA). All of the models cover the adsorption–desorption mechanisms and were primarily based on the char-steam reaction in eq 7, which is relevant to this work.^28^
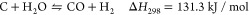
7Table 4 shows a simple explanation of each model with their respective surface reactions.^26^ C(*X*)_*n*_ represents a surface complex comprising a single molecule of C and *n* molecules of *X*. C_f_ refers to a free-active site of carbon on the char surface.

**Table 4:** Three Langmuir–Hinshelwood-Based Mechanism Models Used in This Study

model	surface reaction	remark
oxygen exchange (OE)	C(O) + H_2_ → C_f_ + H_2_O	reverse reaction of the formation of the C(O) complex from C_f_ and steam
associative hydrogen adsorption (AHA)	C_f_ + H_2_ ⇋ C(H)_2_	formation of the C(H)_2_ complex that leads to the H_2_ inhibition effect
dissociative hydrogen adsorption (DHA)	C_f_ + 0.5H_2_ ⇋ C(H)	formation of the C(H) complex that leads to the H_2_ inhibition effect

The OE and AHA mechanisms share the same form of rate model, which is expressed in eq 8. The DHA mechanism has a slightly different rate expression in eq 9 as a result of the non-singular stoichiometric coefficient of H_2_, which is 0.5. Both rates are simply expressed as functions of the hydrogen partial pressure, making it feasible to correlate the presence of hydrogen to its effect on the char conversion rate through adsorption–desorption mechanisms. Here, *a* and *b* can be understood as the representative kinetic parameters, which can further be translated to more comprehensible kinetic parameters, such as rate constants on the corresponding surface reactions shown in Table 3. However, it was rather challenging to do so without any variation in steam and H_2_ concentrations, which means that the parameters *k*_H_2_O_ and *k*_H_2__ were simply unobtainable; thus, only *a* and *b* are presented in this work.
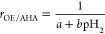
8
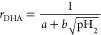
9The hydrogen partial pressure in the rate expression was the arithmetic average of the hydrogen partial pressure on the up- and downstreams of the reactor over the 3 repeated cycles.^29^ The ratio of CO/CO_2_ was understood as the reduction potential and defined as the ratio between the total molar released CO and CO_2_, also over the 3 cycles. Both the average hydrogen partial pressure and reduction potential were obtained from the same data range with that of the gas–solid reaction rate.

### 2.7 Characterization

SEM/EDX JEOL 7800F Prime was used to analyze the surface morphology and elemental distribution of both fresh and used ilmenite samples, which were embedded in epoxy to expose the cross-section surface area under the analysis. XRD Bruker D8 was used to identify the relevant crystalline phases in each ilmenite sample.

## 3 Results and Discussion

### 3.1 Gas Concentration and Mass Conversion Degree

**Figure 3:**
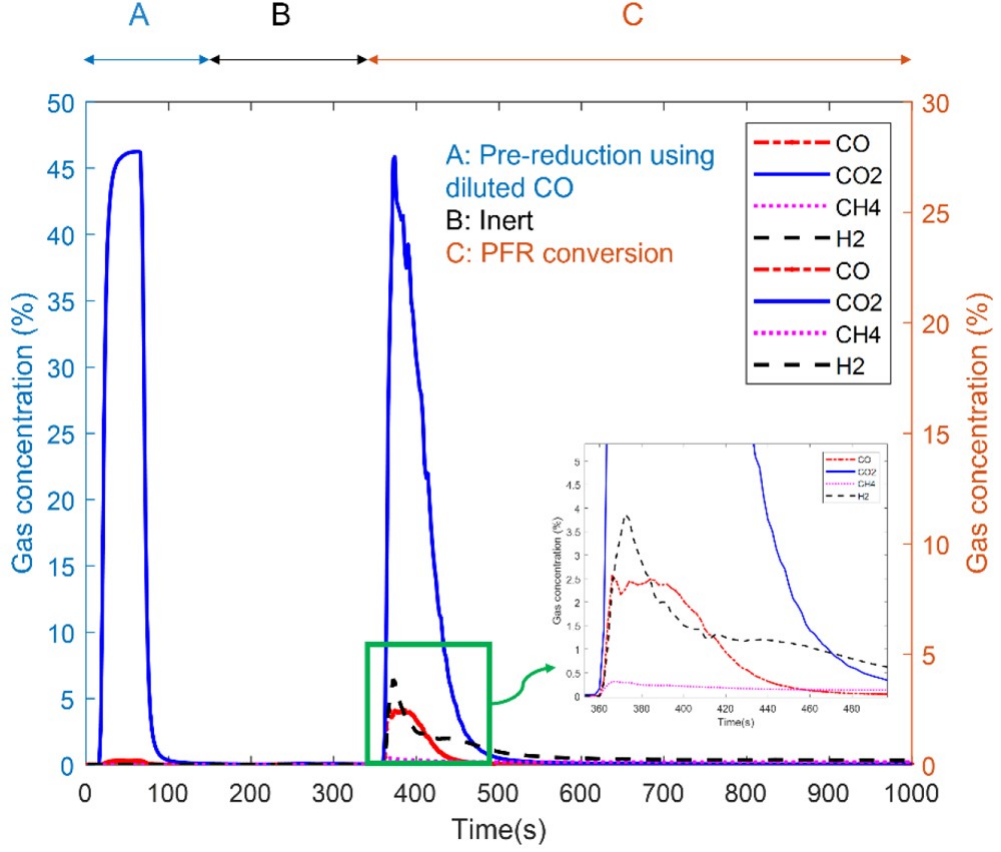
Typical gas concentration during diluted CO injection followed by PFR char conversion with an inert period in between. In this case, the duration of diluted CO injection was 50 s and the reaction temperature was 975 °C. Regions A + B and C follow the left and right axes, respectively. The concentrations of CO, H_2_, and CH_4_ during PFR conversion are enlarged in the onset.

In this study, ilmenite was first exposed to diluted CO injection, which reduced ilmenite to certain extents prior to the conversion of PFR char as solid fuel. This was performed to obtain different known mass conversion degrees of ilmenite prior to the char conversion experiments, thus making it easier to grasp how different reduction degrees may affect the char conversion itself. Figure 3 shows a typical concentration plot during CO injection and solid fuel conversion with an inertness between them over time.

The mass conversion degrees as a result of diluted CO injection are shown in Table 5. Fully oxidized ilmenite was also investigated directly with solid fuels and referred to as a CO injection of 0 s in the table. The average values of the mass conversion degree over temperatures are also provided.

**Table 5:** Mass Conversion Degrees after Pre-reduction with Diluted CO (50:50, CO + N_2_), in Percentage (%)

	temperature (°C)				
duration of diluted CO injection (s)	850	900	950	975	average mass conversion degree (%)
0	0	0	0	0	0
50	0.7	0.6	0.7	0.7	0.7
100	1.4	1.3	1.5	1.4	1.4
150	2.1	2.1	2.2	2.2	2.1
200	2.8	2.7	2.9	2.9	2.8

### 3.2 Arrhenius Parameters

**Figure 4:**
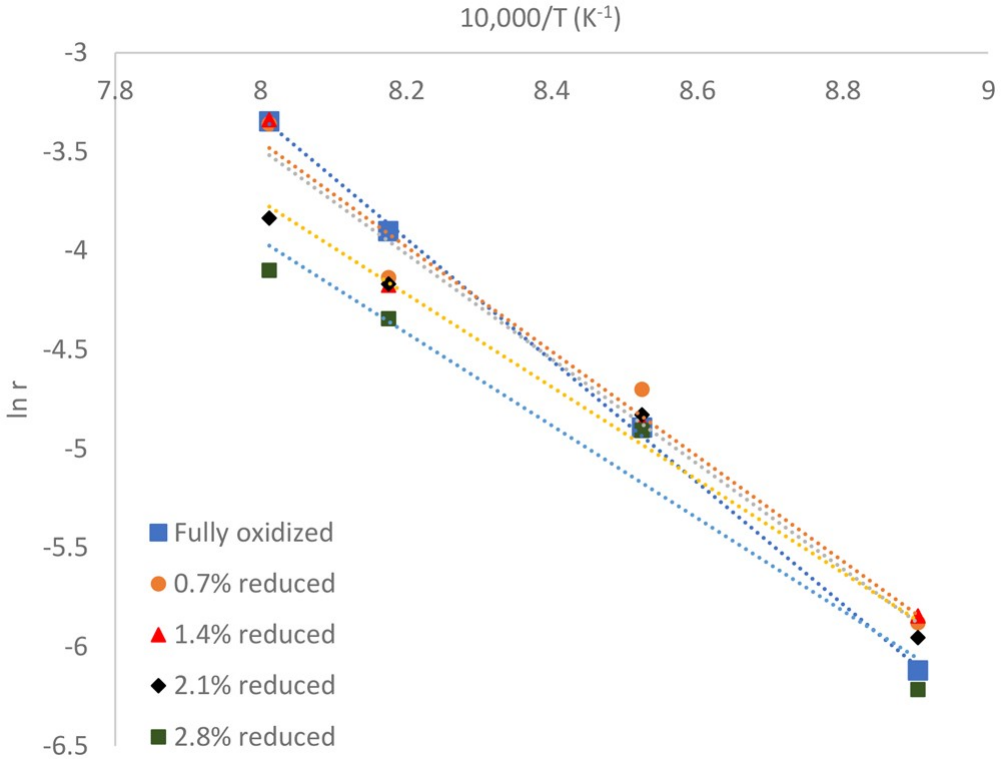
Char conversion rate *r* (in logarithmic form) plotted as a function of the inverse of the temperature (10 000/*T*) according to the Arrhenius equation.

The rate constant *k* and activation energy *E*_a_ of the char conversion could be obtained by making a plot according to eq 6. The plots are shown in Figure 4, and the Arrhenius parameters are provided in Table 6. Here, the average mass conversion degree of ilmenite (see Table 3) was used to show how the parameters between fully oxidized and reduced ilmenite differ.

**Table 6:** Arrhenius Parameters for PFR Char Conversion with Ilmenite at Various Mass Conversion Degrees

parameter	fully oxidized	0.7% reduced	1.4% reduced	2.1% reduced	2.8% reduced
*k* (s^–1^)	2 × 10^9^	5 × 10^7^	5 × 10^7^	3 × 10^6^	3 × 10^6^
*E*_a_ (kJ/mol)	255.66	220.02	220.31	194.96	194.46

It can be seen that the Arrhenius parameters do not differ significantly over the mass conversion degrees. This suggested that the mass conversion degree might not give a major contribution to the char conversion rate. Still, a change in the mass conversion degree could lead to a change in the hydrogen partial pressure, which, in turn, can slow the conversion rate (see section 3.5). This could mean that the mass conversion degree may give an indirect effect to the char conversion rate, particularly when the oxygen carrier, in this case ilmenite, is substantially reduced. An exaggerated reduction does not commonly occur in a larger scale CLC. However, it should be noted that oxygen carrier particle beds may be locally reduced to a higher extent in several processes that involve partial oxidation of fuel, such as in CLG or CLR. This may still cause partial agglomeration and even defluidization; thus, this finding is still crucially applicable to the implementation of a bigger chemical looping process.

### 3.3 Hydrogen Partial Pressure

**Figure 5:**
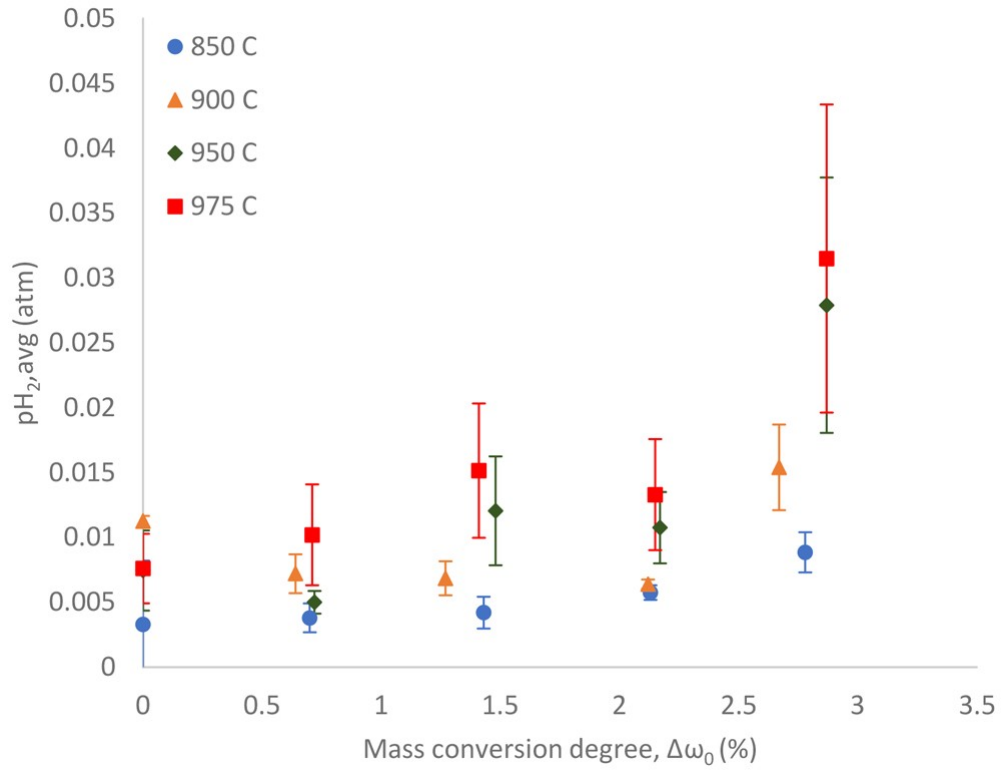
Average hydrogen partial pressure during the char conversion as a function of the mass conversion degree set by pre-reduction with diluted CO.

To examine the hydrogen inhibition effect on the char conversion, the hydrogen partial pressure needs to be obtained first. The average values of the hydrogen partial pressure during the char conversion from the 3 repeated cycles were plotted as a function of the mass conversion degree, which was set by pre-reduction with diluted CO, in Figure 5. A higher mass conversion degree change (Δω_0_) means a lower oxidation degree, which implies a further reduction of ilmenite.

It is important to consider that the average hydrogen partial pressures are quite low, i.e., less than 4% for all investigated cases. This is due to the rather fast reaction between hydrogen and ilmenite.^30^ Still, the graph shows that the more reduced the oxygen carrier preliminary by diluted CO, the higher the hydrogen partial pressure during the char conversion. This trend can be clearly seen at 950 and 975 °C, while that at 850 and 900 °C is inconclusive because the hydrogen concentration did not change significantly. The increasing hydrogen partial pressure was likely caused by the shift in the reaction equilibrium of the water–gas shift reaction,^31^ shown in eq 10.
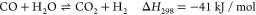
10The more reduced the oxygen carrier, the less oxygen was available during the char conversion, which led to an increase of CO. This subsequently shifted the reaction equilibrium to the right, thus increasing the hydrogen partial pressure.

### 3.4 Reduction Potential

**Figure 6:**
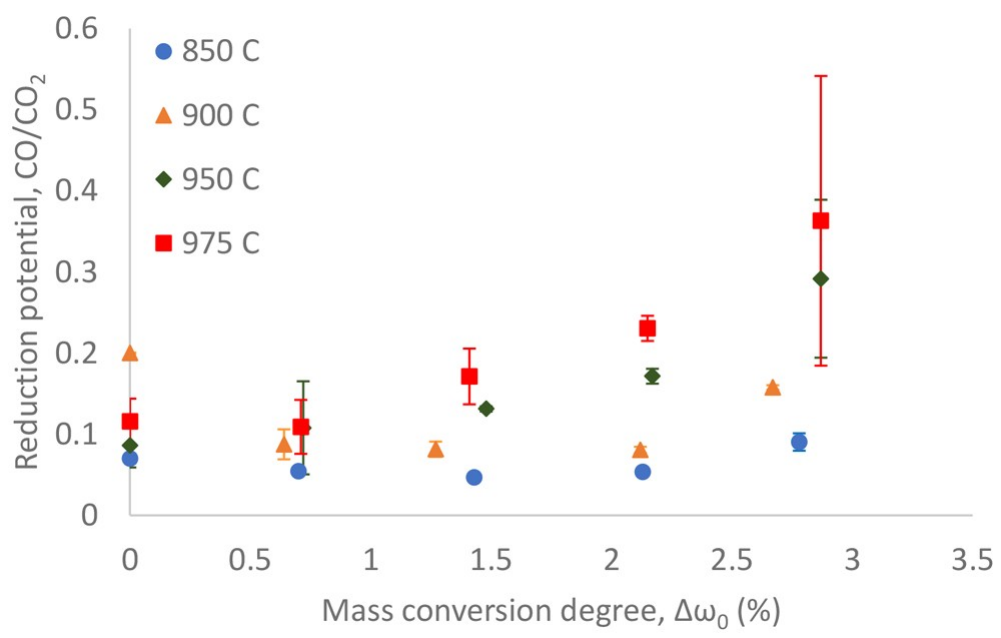
Reduction potential under PFR char conversion plotted as a function of the mass conversion degree of ilmenite set by the pre-reduction with diluted CO.

The CO/CO_2_ ratio during the solid fuel conversion is considered here as the reduction potential. The reduction potential for every mass conversion degree, which was set by the pre-reduction with diluted CO, is shown as a plot in Figure 6. The error bars indicate the standard deviation of the average reduction potential obtained from 3 repeated cycles. Note that some error bars might be not visible in the graph because they are too small compared to the others.

The reduction potential during solid fuel conversion showed an increasing trend as ilmenite was reduced further prior to the gas–solid reaction, particularly at 950 and 975 °C. The increases at 850 and 900 °C were not substantial enough, considering that the reduction potential did not change so much over different mass conversion degrees. This means that, when ilmenite was substantially reduced at higher temperatures of 950 °C and above, the consumption of CO during char conversion was less compared to that at lower temperatures. This is likely due to the lower rate of the CO–ilmenite reaction caused by the hydrogen inhibition effect, which will be explained in the next section. The high uncertainties observed at higher temperatures of 950 and 975 °C at Δω of around 2.8% were probably due to the higher fluctuations of CO and CO_2_ concentrations at such conditions, which caused a higher variation on the calculated CO/CO_2_ ratio.

### 3.5 Char Conversion Inhibition

**Figure 7:**
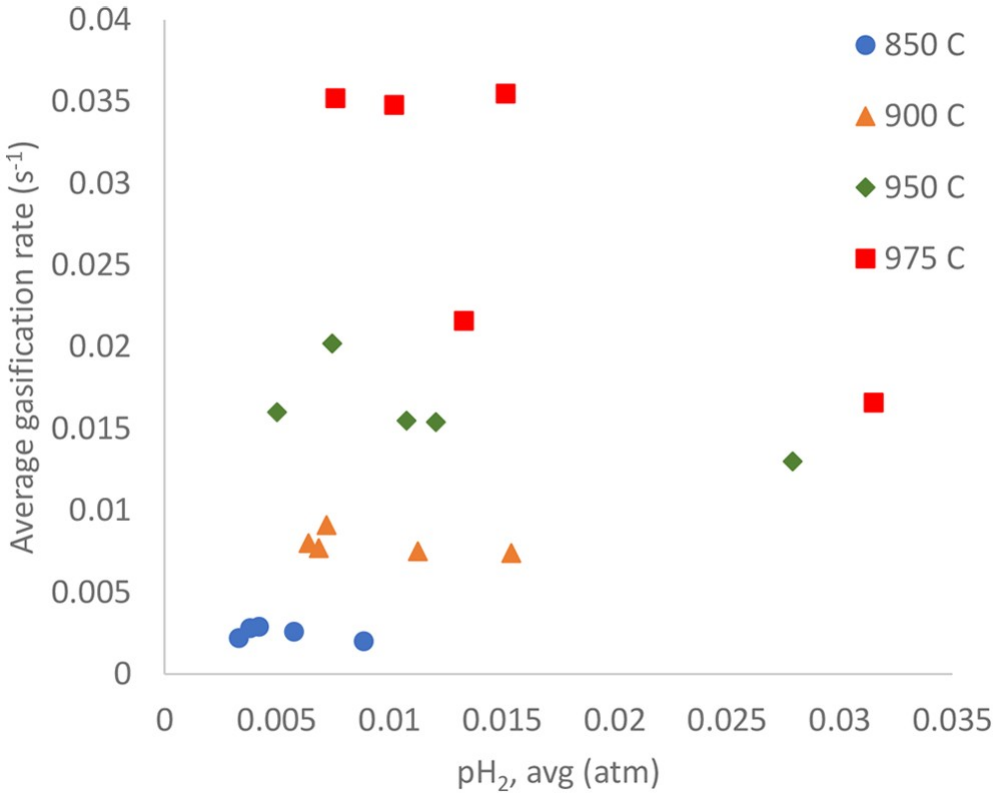
Average conversion rate of PFR char as a function of the hydrogen partial pressure during the char conversion.

A higher hydrogen concentration may slow the char conversion rate substantially.^32^ In section 3.3, it has been shown that a lower mass conversion degree can enhance the hydrogen production. Here, the char conversion rate was plotted as a function of the corresponding hydrogen partial pressure, as shown in Figure 7.

The char conversion rate in Figure 7 does not show a clear declining trend, except at 975 °C. This could be due to the fairly low corresponding hydrogen partial pressure values, less than 4% in all cases. The reason was likely the small quantity of PFR char that was inserted into the reactor, i.e., 0.1 g of char compared to 20 g of ilmenite; therefore, this was probably not enough to confirm the hydrogen inhibition. In this setup, it was difficult to increase the amount of inserted fuel as a result of the possibility of fuel being stuck in or even blocking the feeder.

**Figure 8:**
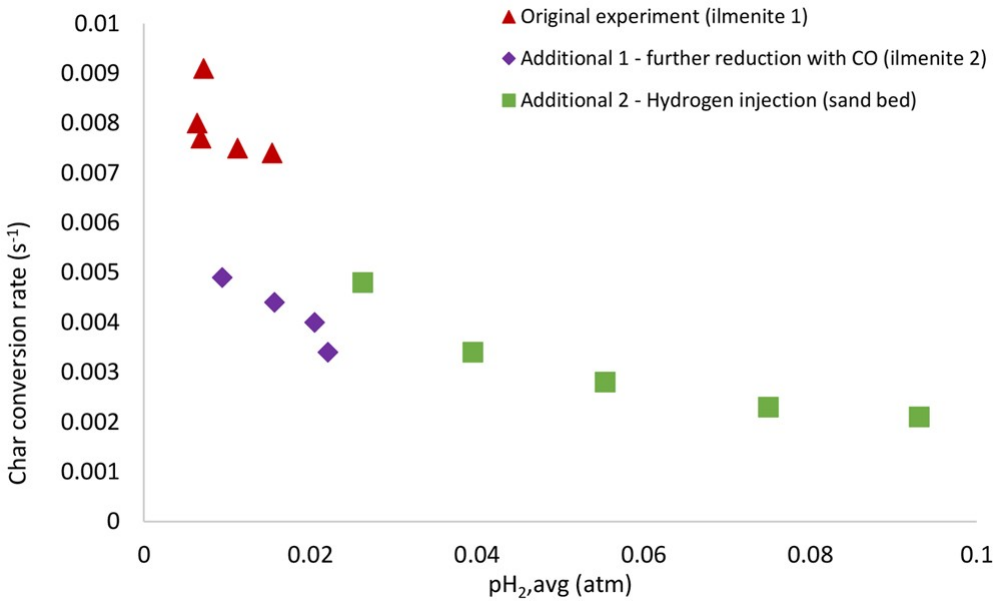
Char conversion rate plotted as a function of the average hydrogen partial pressure for both original and additional experiments at 900 °C with different batches of bed.

To investigate the effect of this limitation, two additional types of experiments were performed according to Table 3. The char conversion rate was then plotted against the average hydrogen partial pressure for both the experiments with ilmenite and sand, while the latter involves hydrogen injection. To have an agreement of the terms, these investigations are called additional, while the previous investigations are called original. Note that all of the additional experiments were performed only once because they were complementary to confirm the hydrogen inhibition effect. Figure 8 shows both the original and additional experiments performed at 900 °C.

In comparison to the original experiments, the char conversion rate was clearly suppressed during the additional experiments as more hydrogen was present in the particle bed. The additional hydrogen injection to the sand bed increased the hydrogen partial pressure, which subsequently gave a substantially lower gas–solid reaction rate. Note that ilmenite used in the original and additional experiments is not the same; i.e., the former might have been activated much more than the latter. This is because every cycle in the additional experiments was only performed once at each temperature, while that in the original experiments was performed 3 times at four temperatures. This explains why the char conversion rate between different experiments may look wildly different at the same hydrogen partial pressure. Still, the decreasing trend on the char conversion rate can be clearly seen. On the other hand, the dilution factor in the batch reactor was rather high, and the hydrogen partial pressures were calculated as arithmetic average values. These explain why the average hydrogen partial pressures still did not exceed 10% of the total pressure, which was atmospheric, even when additional hydrogen was injected. Nevertheless, it is clear that the char conversion rate was affected by the increasing hydrogen partial pressure. Therefore, the hydrogen inhibition effect on the char conversion rate was confirmed.

### 3.6 Mechanism Model Validation

Now that the char conversion inhibition by hydrogen had been confirmed, the next aim was to find the mechanism behind the inhibition. The focus of this study was to find the inhibition mechanism caused by the change in the mass conversion degree; thus, the additional experiment results were excluded in the model fittings because it involved the sand bed particle, for which the mass conversion degree is irrelevant.

**Figure 9:**
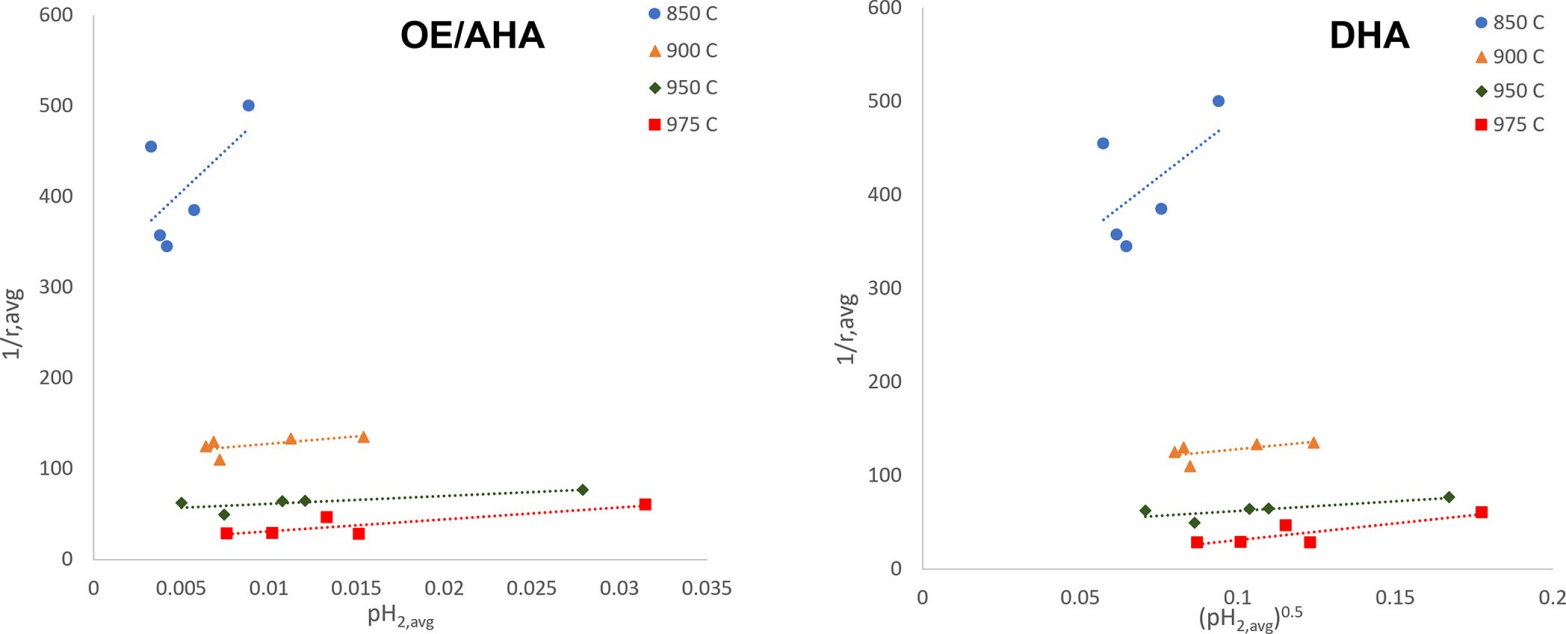
Plotting of Langmuir–Hinshelwood-based model mechanisms, OE/AHA and DHA, based on eqs 8 and 9, respectively, to obtain char conversion kinetic parameters.

As mentioned earlier in section 2.6, the Langmuir–Hinshelwood mechanism models are suitable to interpret the steam char conversion in this work. To obtain the kinetic parameters *a* and *b* (see eqs 8 and 9), it was easier to plot the inverse of the char conversion rate, 1/*r*, as a function of the hydrogen partial pressure. For the dissociative hydrogen adsorption mechanism, however, the domain was the square root of hydrogen partial pressure as a result of the nonlinear order in the reaction mechanism. The fittings can be seen in Figure 9, and the parameters are summarized in Table 7.

**Table 7:** Kinetic Parameters Obtained for Three Langmuir–Hinshelwood Mechanisms

		parameter			
model	rate formula and unit	850 °C	900 °C	950 °C	975 °C
OE/AHA	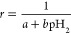 *a* in s and *b* in s atm^–1^	*a* = 313.6	*a* = 111.4	*a* = 52.6	*a* = 17.8
		*b* = 18296	*b* = 1621	*b* = 876	*b* = 1325
DHA	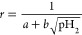 *a* in s and *b* in s atm^–0.5^	*a* = 224.5	*a* = 95.2	*a* = 41	*a* = −4.6
		*b* = 2597	*b* = 329.6	*b* = 211	*b* = 356

One way to check the validity of these models is to plot them against the experimental results. A low hydrogen partial pressure may make it difficult to see the difference between the models, i.e., OE/AHA and DHA; therefore, for this sake, it is more useful to validate the model against both the original experimental data and even the additional experimental data, where additional hydrogen injection was involved.

**Figure 10:**
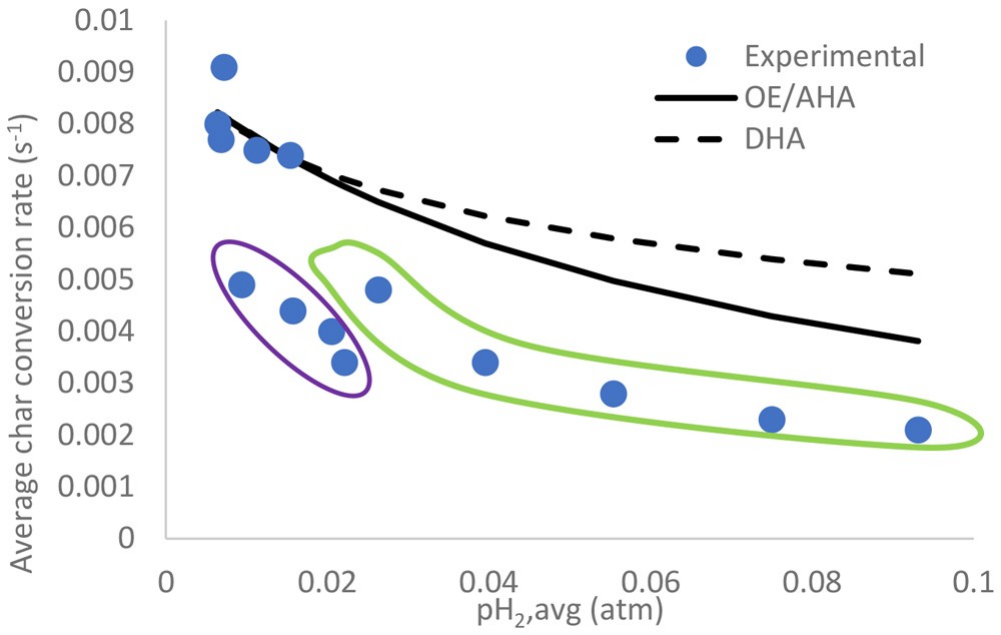
Validation of Langmuir–Hinshelwood models against the experimental data, including the additional experiments, at 900 °C. The further CO reduction and additional hydrogen injection are indicated as dots within the purple and green loops, respectively.

Figure 10 shows that the models fit when the conversion rates were higher during the original experiments but tended to overestimate the rates when the conversion rate went lower as a result of either further CO reduction or hydrogen injection. Still, the OE/AHA models seemed to fit the data better than the DHA model. The substantial differences between rates in similar average hydrogen partial pressure should be taken with consideration that the material used in the additional experiments with further CO reduction was not the same as the original material; i.e., a new batch of unused ilmenite was used in the additional experiments that involved longer reduction periods (250–400 s; see Table 3). Excluding the activation steps, the material used in the original experiments have certainly undergone more oxidation–reduction cycles, i.e., 60 cycles, compared to the material used in the additional experiments, which only involved 4 cycles. This could make a difference on the porosity of ilmenite, the fuel reactivity toward ilmenite, and consequently, the conversion rate itself, because any oxygen carrier material constantly activates itself over more cycles. Moreover, the material used for the experiments involving additional hydrogen injection was not even ilmenite but merely quartz sand (see Table 3). The motivation of using sand has been addressed in the previous section. Apart from these, there could be some little unaccounted factors, such as a different temperature gradient in the reactor and the varying fractions of char that were actually converted. Still, the results demonstrated that the OE/AHA mechanism was likely the relevant mechanism in the gas–solid reaction inhibition by hydrogen.

Determining which mechanism between oxygen exchange and associative hydrogen adsorption that governs the char conversion inhibition is not so straightforward because both mechanisms share the same rate formula. However, Lussier et al.^32^ previously experimented with an annealed char in temperature-programmed desorption (TPD) and reported that all adsorbable hydrogen had already been consumed at 727 °C; thus, hydrogen adsorption is unlikely to take place in a higher temperature. The char used in this work is different, yet the mentioned finding is still deemed relevant to this study. All of the temperatures used in this study were higher than 727 °C; therefore, the possibility that associative hydrogen adsorption controlled the inhibition could be ruled out. Therefore, the oxygen exchange mechanism was likely the most reasonable mechanism that contributed to the gas–solid reaction inhibition by hydrogen.

### 3.7 Characterization

#### 3.7.1 Analysis of Crystalline Phases

**Figure 11:**
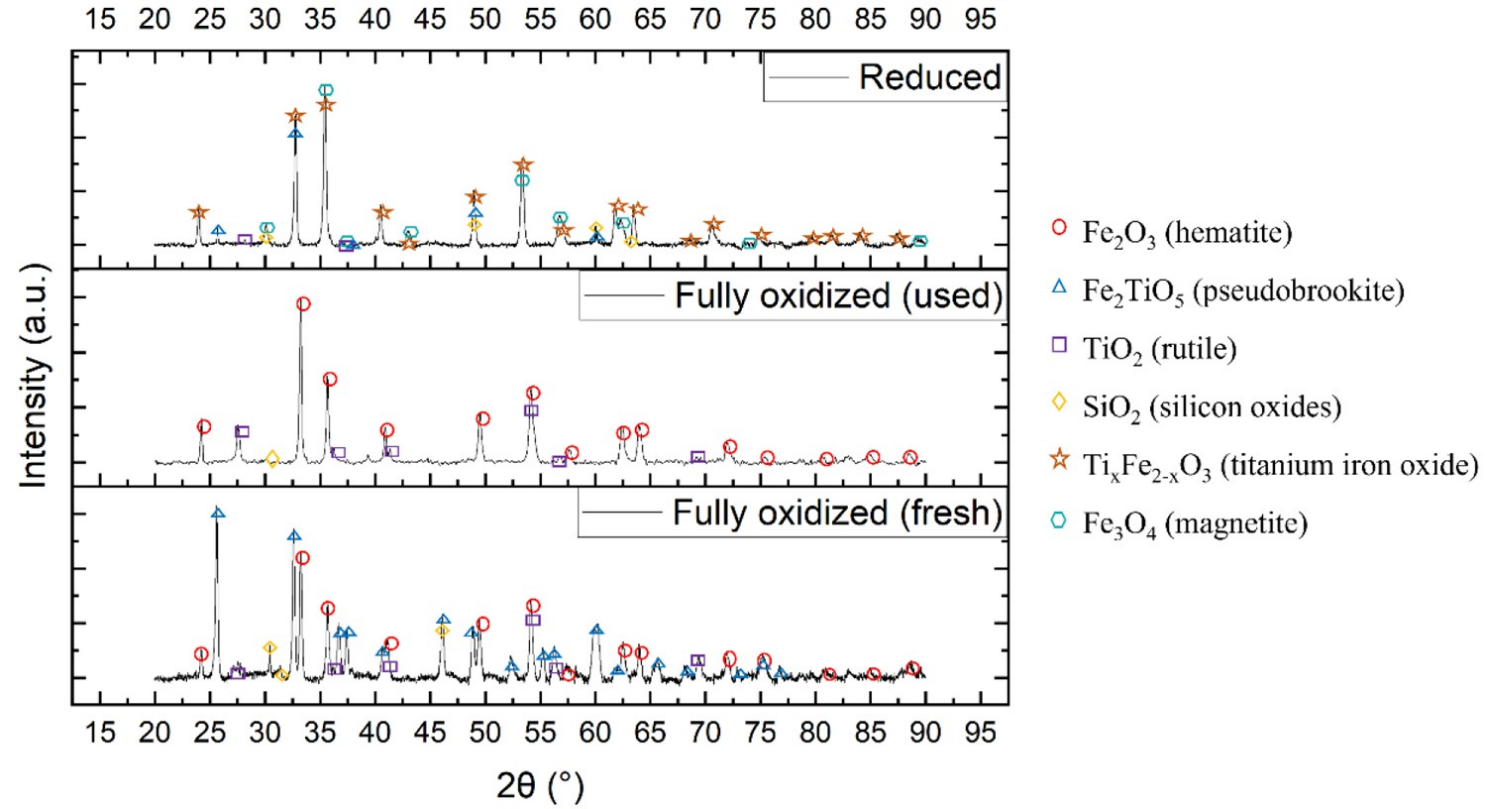
Diffractogram of ilmenite in three states: fully oxidized (fresh), fully oxidized (used), and reduced.

The crystalline phases of ilmenite before and after the experiments, which can be referred to as fresh and used samples, respectively, were identified by XRD with the aim to obtain some information about its phase transformation. The fresh sample was in a fully oxidized state, while the used sample was available and analyzed in two oxidation states: fully oxidized and reduced. Figure 11 comprises three diffractograms: fully oxidized (fresh), fully oxidized (used), and reduced. The used sample underwent 60 cycles of oxidation–reduction, excluding the activation steps.

Hematite (Fe_2_O_3_) and pseudobrookite (Fe_2_TiO_5_) were the main phases in fully oxidized ilmenite, and this has been previously reported as general information for ilmenite.^33^ Titanium oxide was also present as the phase of TiO_2_ (rutile). This was not unexpected because pseudobrookite may be decomposed into hematite and rutile at low temperatures.^34^ Fully oxidized used ilmenite comprised mainly only hematite (Fe_2_O_3_) and rutile (TiO_2_). In reduced ilmenite, the phases of magnetite (Fe_3_O_4_) and titanium iron oxide (Ti_*x*_Fe_2–*x*_O_3_) were detected. Both phases were known as the reduced forms of hematite and pseudobrookite, respectively. A low intensity of pseudobrookite, however, was also still present. This is interesting because the phase of pseudobrookite was not detected in the fully oxidized used sample, thereby indicating a very low intensity at such a state. The peaks of pseudobrookite might have been overlapped by the much stronger intensity of hematite. The phase of rutile (TiO_2_) did not reduce to another titanium oxide phase; this was in line with a previous study reporting that TiO_2_ would not reduce before the formation of elemental iron,^35^ which was not observed here. This demonstrates that, even if ilmenite was in a reduced state and had undergone multiple oxidation–reduction cycles, the formation of wüstite or elemental iron, which can cause defluidization,^1^ did not take place here. Therefore, the use of ilmenite in CLG using biomass char as fuel can be expected to experience no significant problem with respect to the fluidization performance, even if the material was reduced to higher extents compared to that in the FR in CLC.

#### 3.7.2 Surface Topography and Elemental Distribution of Ilmenite

**Figure 12:**
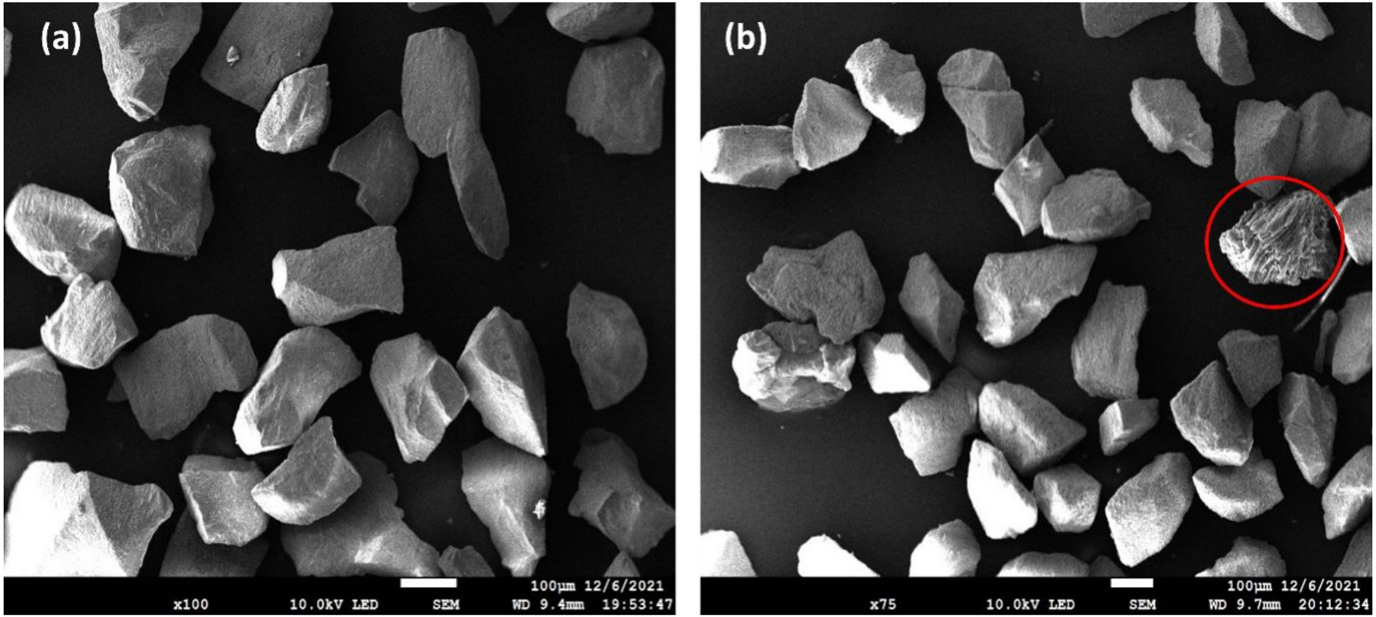
Surface topography of ilmenite particles visualized by SEM at (a) fully oxidized (fresh) and (b) reduced states. The red circle indicates an ash-like substance.

**Figure 13:**
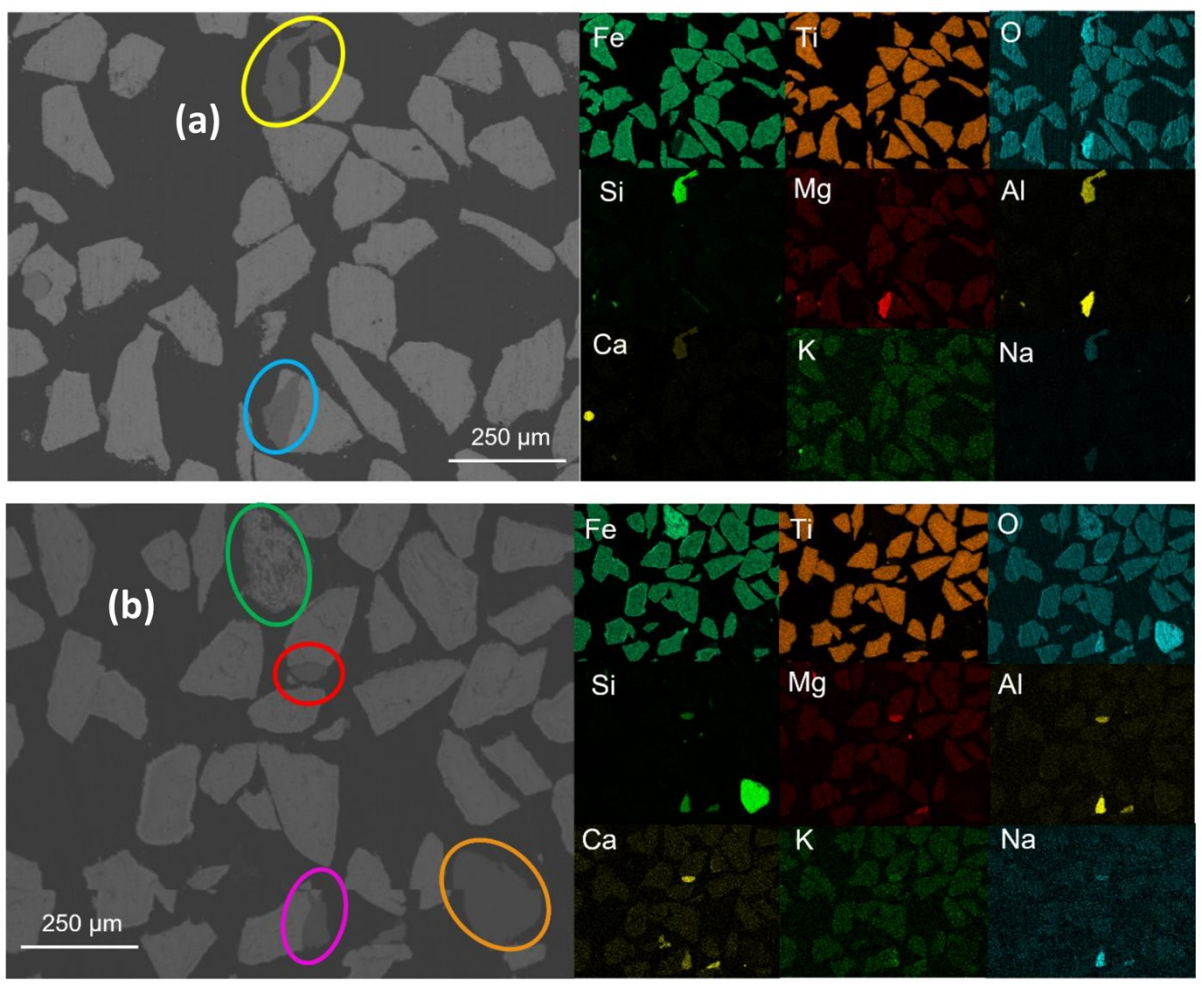
Elemental distribution in ilmenite particles at (a) fully oxidized (fresh) and (b) reduced states. Explanations about the circles: (i) the yellow circle indicates a contaminant in the fresh sample; (ii) the blue circle indicates a high intensity of Mg and Al; (iii) the green circle indicates a titanium-free particle, (iv) the orange circle indicates a particle that contains only silicon oxide; and (v) the violet and red circles indicate two areas that contain a high intensity of alkali substances.

The surface topography and elemental distribution of ilmenite before and after the experiments, i.e., oxidized fresh and used reduced, respectively, were examined using SEM/EDX. Figures 12 and 13 visualize the surface of particles and cross-sectional area of ilmenite, respectively. The oxidation states for these samples are still the same case as that mentioned in the previous section; i.e., the particles were fully oxidized before the experiments and became subsequently reduced afterward. The reduced samples had undergone 60 cycles, excluding the activation steps.

In Figure 12, in comparison to the fresh sample before the experiment, neither agglomeration nor visible major surface erosion was observed under SEM because the particles still have pointy edges even after the experiment, i.e., in the reduced state. Still, the surface of reduced ilmenite has lost the rough texture that it once had in the fresh sample. There is an ash-like substance seen in the reduced sample indicated within the red circle, which likely originated from the PFR char.

In Figure 13, iron was distributed quite evenly in the fully oxidized fresh sample. A contaminant was also detected under SEM/EDX, indicated here with a yellow circle. The substance showed a high intensity of silicone and aluminum and a low intensity of calcium and sodium. Oxygen in this substance showed a higher intensity than that in other particles. Additionally, the part of an oxygen particle indicated in the blue circle also showed a high intensity of magnesium and aluminum, even probably a small amount of sodium. It seemed that oxygen in this area also showed a higher intensity compared to that coupled to iron and titanium. This demonstrates that even the fresh fully oxidized ilmenite was not completely free from contaminating substances, which is reasonable considering that the oxygen carrier was obtained in an ore form.

In the reduced sample after the experiment, iron formed an outer layer of the particles; this migration of iron from the particle core to the outside layer has been reported previously.^36^ A single titanium-free iron oxide and pure silicon oxide particle were detected; see the green and orange circles. The area within the violet circle, which is attached to the oxygen carrier particle, showed high intensities of sodium, aluminum, silicon, and calcium and an even higher intensity of oxygen compared to that in the other particles. This situation was also seen in the fresh unused sample; thus, there is nothing strange here. Interestingly, the same area also showed a slightly higher intensity of potassium, which was not seen in the fresh sample. The same phenomenon was also seen inside the red circle. Ilmenite has been previously reported to be able to interact with potassium, where iron in reduced ilmenite migrated to the outer layer and reacted with potassium to form K titanate.^37−39^

These findings clearly show that ilmenite has been reduced during the experiments; this can be seen from, among the others, migrating iron to the outer surface. Despite this, there was no agglomeration seen on the particles, and no bed defluidization was observed either. Therefore, even when ilmenite was reduced prior to the char gasification, which can happen in CLG, the material will likely not experience such mentioned issues. The use of ilmenite in CLG for multiple cycles can therefore be recommended according to these findings with respect to the physical performance of ilmenite.

## 4 Conclusion

This study aimed to examine the effect of the mass conversion degree on solid fuel conversion in a fluidized bed, because a substantial change in the mass conversion degree is expected in a CLG process compared to normal CLC. Dependent upon the mass conversion degree of ilmenite, the activation energy of PFR char with ilmenite as the oxygen carrier ranged from about 194 to around 256 kJ/mol. It was found that the lower the mass conversion degree of the oxygen carrier, the higher the reduction potential and the hydrogen partial pressure in the bed. This eventually led to a lower char conversion rate, which is known as the inhibition effect caused by hydrogen. Therefore, the change in the mass conversion degree had an indirect effect on the char conversion rate. Interpretation using Langmuir–Hinshelwood models suggested that the oxygen exchange mechanism likely took place under char conversion. Characterization with XRD confirmed that ilmenite was reduced during the fuel conversion without the formation of either wüstite or elemental iron, which may cause bed defluidization. Observation under SEM/EDX suggested that potassium reacted with migrating iron on the surface of ilmenite particles. The characterization results were encouraging because there was no agglomeration seen, despite the substantial reduction and multiple cycles to which the material had been exposed. No bed defluidization was observed during the whole experiments. Therefore, the use of ilmenite in CLG using biomass char will likely not cause any major issues with respect to the agglomeration and fluidization performances.

## Abbreviations Used

AR: air reactor
AHA: associative hydrogen adsorption
BCLG: biomass chemical looping gasification
CLC: chemical looping combustion
CLG: chemical looping gasification
CLR: chemical looping reforming
DHA: dissociative hydrogen adsorption
*E*_a_: activation energy (kJ/mol)
FR: fuel reactor
*k*: fuel conversion rate constant (s^–1^)
*M*_*i*_: molecular weight of element *i* (g/mol)
*m*_ox_: mass of a fully oxidized sample (g)
*ṅ*: gas molar flow (mol/s)
OE: oxygen exchange
PFR: pine forest residue
pH_2_: hydrogen partial pressure (atm)
*r*: gasification rate
*t*: time (s)
TPD: temperature-programmed desoprtion
*X*_c_: fraction of char conversion
*x*_*i*_: molar fraction of species *i*
ω: mass conversion degree
